# Diversity, Distribution and Hydrocarbon Biodegradation Capabilities of Microbial Communities in Oil-Contaminated Cyanobacterial Mats from a Constructed Wetland

**DOI:** 10.1371/journal.pone.0114570

**Published:** 2014-12-16

**Authors:** Raeid M. M. Abed, Samiha Al-Kharusi, Stephane Prigent, Tom Headley

**Affiliations:** 1 Sultan Qaboos University, College of Science, Biology Department, P.O. Box: 36, postal code 123, Al Khoud, Sultanate of Oman; 2 BAUER Resources, Constructed Wetland Competence Centre, P.O. Box 1186, P.C. 114, Al Mina, Muscat, Sultanate of Oman; National Taiwan University, Taiwan

## Abstract

Various types of cyanobacterial mats were predominant in a wetland, constructed for the remediation of oil-polluted residual waters from an oil field in the desert of the south-eastern Arabian Peninsula, although such mats were rarely found in other wetland systems. There is scarce information on the bacterial diversity, spatial distribution and oil-biodegradation capabilities of freshwater wetland oil-polluted mats. Microbial community analysis by Automated Ribosomal Spacer Analysis (ARISA) showed that the different mats hosted distinct microbial communities. Average numbers of operational taxonomic units (OTUs_ARISA_) were relatively lower in the mats with higher oil levels and the number of shared OTUs_ARISA_ between the mats was <60% in most cases. Multivariate analyses of fingerprinting profiles indicated that the bacterial communities in the wetland mats were influenced by oil and ammonia levels, but to a lesser extent by plant density. In addition to oil and ammonia, redundancy analysis (RDA) showed also a significant contribution of temperature, dissolved oxygen and sulfate concentration to the variations of the mats’ microbial communities. Pyrosequencing yielded 282,706 reads with >90% of the sequences affiliated to *Proteobacteria* (41% of total sequences), C*yanobacteria* (31%*)*, *Bacteriodetes* (11.5%), *Planctomycetes* (7%) and *Chloroflexi* (3%). Known autotrophic (e.g. *Rivularia*) and heterotrophic (e.g. *Azospira*) nitrogen-fixing bacteria as well as purple sulfur and non-sulfur bacteria were frequently encountered in all mats. On the other hand, sequences of known sulfate-reducing bacteria (SRBs) were rarely found, indicating that SRBs in the wetland mats probably belong to yet-undescribed novel species. The wetland mats were able to degrade 53–100% of C_12_–C_30_ alkanes after 6 weeks of incubation under aerobic conditions. We conclude that oil and ammonia concentrations are the major key players in determining the spatial distribution of the wetland mats’ microbial communities and that these mats contribute directly to the removal of hydrocarbons from oil field wastewaters.

## Introduction

Wastewater reuse is an essential strategy for the conservation of water resources, particularly in developing countries, which suffer from water scarcity throughout the year (expected to reach 35 countries by 2025) [1]. Constructed wetlands treatment systems are engineered and designed to exploit natural processes, including those of microbial communities associated with wetland plants and soils, for the cleanup of wastewaters [2]–[4]. These systems are low-cost, self-sustaining, easily operated and have been successfully employed worldwide, over the last six decades, to remove a diverse array of contaminants from municipal, agricultural and industrial wastewaters [2], [5], [6]. In recent years, constructed wetlands have been used for the treatment of residual waters produced from oil fields in order to remove hydrocarbons and to enhance water quality for irrigation purposes and/or safe discharge in aquatic systems [7]–[10]. Research on wetlands was mainly performed to create new designs, monitor changes in pollutant concentrations and wetland plants, study seasonal changes in the treatment performance and assess the quality of produced water and its suitability for reuse in irrigation [7]–[10]. Although microorganisms play a central role in the degradation of pollutants and the biogeochemical transformation of nutrients in wetlands, little is known about their diversity and distribution in comparison to soils and aquatic ecosystems, especially with regard to hydrocarbon degradation.

Produced water is water from underground formations that is brought to the surface via wells during oil and gas production. Even after the majority of the oil and gas has been separated, produced water is typically contaminated with residual hydrocarbons. Globally, it is estimated that approximately 50 million m^3^/day of produced water are generated [11], the management of which represents a large environmental challenge for the oil and gas industry. In 2010, BAUER commissioned one of the world’s largest constructed wetland systems for the treatment of oil-polluted produced water in south-east Arabia. The system consists of more than 700 ha of constructed wetlands and ponds, and is currently used to treat 95,000 m^3^ of oil-field production water every day. Field observations of the wetland showed that large areas of the wetland soils are covered with well-developed photosynthetic cyanobacterial mats, although such mats were rarely reported for wetlands [12]–[14]. These mats showed differences in appearance, texture, ambient oil concentrations and the surrounding density of wetland plants. In addition to exposure to oil, the mats experience intense sunlight and temperatures that may exceed 50°C in hot summers. Hence, it was expected that these constructed wetland mats harbor extremophilic microorganisms and that microbial degradation of hydrocarbons by these mats contribute, among other processes, to efficient remediation of the oil-polluted waters. Indeed, previous research has shown the ability of marine cyanobacterial mats to develop well on oil polluted sediments and demonstrated their ability to degrade hydrocarbons under aerobic and anaerobic conditions [15], [16]. Cyanobacteria and aerobic oil-degrading bacteria in mats constituted an ideal consortium for the degradation of oil components [17], [18].

The aim of this investigation is threefold: 1) to study the α (local richness) and β (community turnover) diversity of microbial communities in 15 different cyanobacterial mats in the produced water treatment wetlands, 2) to study the spatial distribution of microbial communities and how it is influenced by varying environmental conditions, such as oil contamination level, nutrient concentrations and plant density, and 3) to investigate the oil degradation capability of mat microorganisms.

## Materials and Methods

### Sampling Sites and Nutrient Analysis

The site where samples were collected is part of a facility owned and operated by the BAUER company. The necessary permission to access the site was acquired from BAUER, who are a co-author of this publication. The field studies did not involve any endangered or protected species. The specific location of the study cannot be disclosed for reasons of commercial confidentiality. Tom Headley of BAUER Resources should be contacted for future permission to access the site.

**Figure 1:**
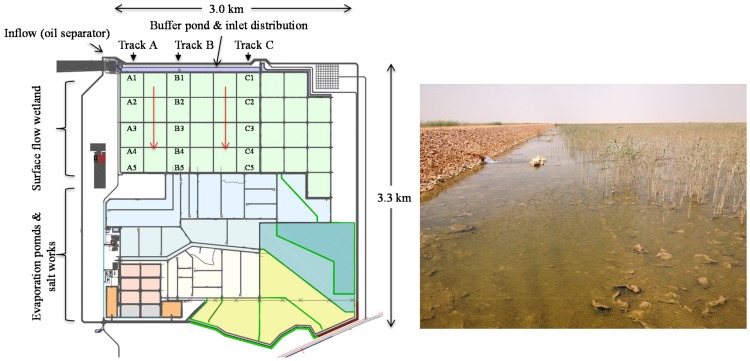
The layout of the produced water treatment plant and wetland sampling locations (left) and a photograph depicting the wetland and the cyanobacterial mats covering the sediments (right).

The layout of the produced water treatment system includes a pipeline entering the system and leading to an oil and water separator (Fig. 1). The water is then distributed by gravity feed from a buffer pond to 350 ha of surface flow constructed wetlands divided into nine parallel streams (“Tracks”) of four cells in series. Overall, the wetland consists of 36 ten-hectare cells. The wetland is planted with a mixture of wetland plant species, including *Phragmites australis*, *Typha domingensis* and *Scoenoplectus littoralis*. Treated water from the wetland facility is then channelled to 350 ha of evaporation ponds to recover salts, which can be reused for drilling operations in oilfields. A portion of the wetland-treated produced water is also reused for drilling operations and for irrigation of halophytic plants. Cyanobacterial mat samples were collected from Tracks A, B and C (Fig. 1) in May 2013, in order to span a range of oil exposure levels and plant densities. Within each of the monitored tracks, samples were collected from the inlet zone of each of the four wetland cells in series and from the outlet zone of the 4^th^ cell in each track (numbered from 1 to 5 for each track, Fig. 1). This sampling pattern was designed to span the range of water quality and plant densities throughout the wetland, as there is a depletion of nutrients and hydrocarbons, increase in salinity and decrease in plant density as you move from the 1^st^ to the 4^th^ cell in each track. From each sample location, three samples were collected, stored in sterile plastic boxes and transferred to the laboratories in a cool box, where they were immediately stored at −20°C until analysis.

Physical water quality parameters including water temperature, dissolved oxygen, pH, oxidation reduction potential (ORP) and conductivity were measured on site after collection of samples using a calibrated portable multimeter (WTW Multiline P4 Universal Meter and Hach HQ30d Flexi Meter). For the analysis of chemical parameters, 400 ml water samples were collected form each sample location in 500 ml bottles. A fraction of the water sample was preserved in 10% zinc acetate for the estimation of sulfide. Oil in water, ammonia (NH_3_^–^N), phosphate (P-PO_4_^3−^), sulfate (SO_4_^2−^) and boron (B) were measured in the water samples using a spectrophotometer (DR 3900 spectrophotomer, Hach Lange, Germany) according to Hach standard methods. A qualitative assessment was made of the vegetation cover in the vicinity of each sample point at the time of sampling. Three vegetation coverage categories were used: highly vegetated (75–100%); moderately vegetated (25–75%) and sparsely vegetated (0–25%).

### Microbial Community Analysis by ARISA

ARISA was performed in order to obtain a broad perspective of the variation of microbial diversity in the wetland mats and how this variation is influenced by the different environmental parameters. ARISA allows the analysis of a large number of samples for reliable statistical analysis. 300–500 mg of each cyanobacterial mat sample (3–5 replicates) were subjected to DNA extraction using the PowerBiofilm DNA isolation kit (MOBIO laboratories, Inc., Carlsbad, CA) according to the manufacturer’s instructions. Polymerase chain reaction (PCR) amplification was performed in triplicate for each DNA extract with the same amounts of DNA (as determined by Nanodrop, Thermo Scientific, Germany) using the universal primer ITSF (5′-GTCGTAACAAGGTAGCCGTA-3′) and the FAM-labelled eubacterial ITSReub primer (5′-GCCAAGGCATCCACC-3′) [19]. PCR cycling conditions consisted of an initial denaturation step at 94°C for 3 min, followed by 30 cycles of 94°C for 45 s, 55°C for 45 s, 72°C for 90 s, and a final extension step of 72°C for 5 min. Each PCR reaction contained 1× PCR buffer (Promega, Madison, WI, USA), 2.5 mM MgCl_2_ (Promega), 0.25 mM of 40 mM dNTP mix (Promega), bovine serum albumin (3 µg µl**^−^** ^1^, final concentration), 25 ng extracted DNA, 0.05 units GoTaq polymerase (Promega) and 400 nM each of the forward and reverse primers. The PCR products were purified using Sephadex G-50 Superfine (Sigma-Aldrich, Munich, Germany). One hundred and fifty ng of DNA was then mixed with 0.5 µl of internal size standard MapMarker 1000 ROX (50–1000 bp; BioVentures Inc., Washington, DC, USA) and the amplified fragments were discriminated by capillary electrophoresis on an ABI PRISM 3130×*l* Genetic Analyzer (Applied Biosystems). The ARISA profiles were analyzed using the GeneMapper software v 3.7 (Applied Biosystem, Carlsbad, CA, USA). The total peak area per sample was normalized to one and only fragments between 100–1000 bp were considered. A “fixed window” binning strategy with a bin size of 2 bp was applied to the ARISA data [20], and the binning frame that offered the highest pairwise similarities among samples was subjected to multivariate analyses (see below). An operational taxonomic unit (OTU_ARISA_) was considered present in a given sample only if it was detected at least twice among the three replicated PCRs from the DNA extracts of that particular sample [20].

Statistical analysis of ARISA fingerprints was carried out using the PAST program (Paleontological Statistics, ver. 1.47, http:\\folk.uio.no\ohammer\past) and the R v.2.15.0 statistical platform using the *vegan* package [21]. The consensus ARISA table containing samples by OTUs_ARISA_ was used to calculate pairwise similarities among samples based on Bray-Curtis dissimilarity index. A multivariate analysis of all sites was performed using multidimensional scaling (MDS) based on Bray-Curtis dissimilarities as described in [22] to examine for significant differences in mat communities in different tracks and at different levels of oil pollution, NH_3_^–^N concentrations and plant densities. Ordination of the Bray-Curtis dissimilarities was performed using non-metric MDS, with 100 random restarts, taking into account the presence/absence as well as the relative fluorescence intensity (RFI) of peaks in the ARISA profiles of all samples. The MDS results were plotted in two dimensions. Analysis of similarities (ANOSIM) with Bonferroni corrected *P* values was carried out to test for significant differences between the defined sample groupings. ANOSIM produces a sample statistic (*R*), which represents the degree of separation between test groups [23]. OTU_ARISA_ partitioning was used to find out the number of OTUs_ARISA_ that are specific for each dataset in the MDS analysis and the number of shared OTUs_ARISA_ between different datasets. This was done on ARISA datasets using Microsoft Excel and a custom R script [20].

Canonical redundancy analysis (RDA) was used to investigate the significance of wastewater physical and chemical parameters on the structure of microbial communities in the wetland mats. ARISA profiles were first Hellinger transformed [22] and the effect of different parameters were investigated by canonical variation partitioning [22], where the variation and covariation of these parameters were partitioned into pure and covarying fractions. For each response data model, the most significant variables (i.e. oil in water, NH_3_^–^N, water temperature and SO_4_^2−^) were selected by redundancy analysis using stepwise selection and by minimizing the Akaike Information Criterion. Statistical significances were assessed by 1000 permutation of the reduced models. All statistical calculations were performed with the R statistical platform using the *vegan* and *MASS* packages [24].

### Pyrosequencing and Sequence Analyses

Pyrosequencing was performed to obtain a taxonomic affiliation of major bacterial classes and genera in the wetland mat communities. Purified DNA extracts were submitted to Molecular Research MR DNA Laboratory (Shallowater, TX, USA) for tag-pyrosequencing. Bacterial tag-encoded FLX amplicon pyrosequencing (bTEFAP) was performed as described before [25], [26] using the GS FLX titanium sequencing kit XLR70. One-step PCR was performed using a mixture of hot-start and hot-start high fidelity taq polymerases resulting in amplicons that extend 350–450 bp from the 27F region (*E. coli* rRNA numbering). The bTEFAP sequencing was performed according to the MR DNA protocols (www.mrdnalab.com).

Sequence analysis was primarily performed by the MR DNA laboratory using QIIME pipeline [27], where barcodes and primers were removed, sequences were denoised and Chimers were removed. OTUs based on a 97% sequence similarity threshold (termed hereafter as OTUs_0.03_ to distinguish them from ARISA-based OTUs) were generated. The sequences were taxonomically classified using GreenGenes database [28]. Since different pipelines and algorithms have been shown to result in different taxa assignments [29], [30], we performed an additional analysis using the NGS pipeline of the SILVA rRNA gene database project (SILVAngs) [31]. Each read was aligned using the SILVA Incremental Aligner (SINA) [32] against the SILVA SSU rRNA SEED and quality controlled [31]. Reads shorter than 50 aligned nucleotides and reads with more than 2% of ambiguities, or 2% of homopolymers, respectively, were excluded from further processing. Identical reads were then identified (dereplication), the unique reads were clustered (OTUs_0.03_), and the reference read of each OTU_0.03_ was classified using cd-hit-est (version 3.1.2; http://www.bioinformatics.org/cd-hit) [33]. The classification was performed by a local nucleotide BLAST search against the non-redundant version of the SILVA SSU Ref dataset (release 115; http://www.arb-silva.de) using blastn (version 2.2.22+; http://blast.ncbi.nlm.nih.gov/Blast.cgi) with standard settings [34]. The classification of each OTU_0.03_ reference read was mapped onto all reads that were assigned to the respective OTU_0.03_, yielding the number of individual reads per taxonomic path. OTU_0.03_ number and Chao 1 were calculated for each dataset. For large sequence datasets, the average diversity indices were calculated after performing three randomized selections of 12000 sequences, using a custom script. Reads without any BLAST hits or reads with weak BLAST hits, were assigned to the meta group “No Relative” in the SILVAngs fingerprint. A heatmap was constructed to compare the distribution of different bacterial genera and families between the different samples. SIMPER (similarity percentage) analysis of the sequence data was performed using PAST program to find out the bacterial genera that account for the community differences in the mat samples from different tracks and in relation to the different levels of oil pollution, NH_3_^–^N concentrations and plant densities.

### Biodegradation Experiments

The ability of mats from Track A (i.e. A1-5) to degrade crude oil was tested in 100 ml flask experiments. Each flask received 0.5 g of fresh mat, 0.5% (v/v) crude oil and 20 ml artificial seawater medium (MgCl_2_.6 H_2_O (5.6 gl**^−^** ^1^), MgSO_4_.7 H_2_O (6.8 gl**^−^** ^1^), CaCl_2_.2 H_2_O (1.47 gl**^−^** ^1^), KCl (0.66 gl**^−^** ^1^), KBr (0.09 gl**^−^** ^1^), KH_2_PO_4_ (0.15 gl**^−^** ^1^) and NH_4_Cl (0.2 gl**^−^** ^1^), supplemented with trace elements mixtures and vitamins [35], [36]. The flasks were shaken at 90 rpm and incubated at 30°C under light (2930 Lux intensity). Triplicate treatments were maintained for every sample as well as biotic (oil+dead autoclaved mat) and abiotic (oil only) controls.

Hydrocarbon biodegradation was followed by measuring the concentration of extractable hydrocarbons (mainly C_12_–C_30_ saturated alkanes) using gas chromatography-mass spectrometry (GC-MS) after 6 weeks of incubation. Two grams of mats were extracted 3 times in 10 ml dichloromethane (DCM). The pooled extract was filtered with non-absorbent cotton to remove solid particles and the filtrate was evaporated using a rotary evaporator. Hydrocarbons were analyzed using a GC-MS, equipped with a flame ionization detector and a 30 m×250 µm capillary column (Rtx-5MS) and were quantified using an external standard (C_7_–C_30_). Helium gas was used as a carrier at a flow rate of 1 ml min**^−^** ^1^ and the injector and detector were maintained at 290°C. The oven temperature was programmed from 80°C (initial hold time 2 min) to 290°C (final hold time 30 min) at a rate of 10°C min**^−^** ^1^. The percent degradation of each alkane was calculated by applying the formula (control – treatment)/control*100).

### Nucleotide sequence accession number

Sequence data from this study were submitted to the NCBI Sequence Read Archive (SRA) under the accession number (Bioproject: PRJNA258311).

## Results

### Wastewater Physical and Chemical Characteristics

Oil in water content ranged between 0.1 to 23.6 mg l**^−^** ^1^, with decreasing concentrations from sample points 1 to 5 (wetland inlet to outlet) in the three Tracks A, B and C (Table 1). The water temperature in the different cells showed differences, with values between 26 to 36°C and with slightly warmer waters in Track A than in Track B and C. The highest temperatures were measured in the heavily polluted cells A1, B1 and C1. These cells also had higher nutrient concentrations (NH_3_^−^N and P-PO_4_^3−^), but less dissolved oxygen, compared to the others. pH and conductivity showed an increase from sample points 1 through 5 within each wetland track. The sample locations A1, A3, B2 and C1 exhibited the highest coverage of wetland plants, whereas locations A2, A5, B1 and B5 contained little or no vegetation.

**Table 1:** Physical-chemical water quality characteristics of the different wetland sample locations, where the cyanobacterial mats were collected.

Track	A1	A2	A3	A4	A5	B1	B2	B3	B4	B5	C1	C2	C3	C4	C5
Oil in water (mg l**^−^** ^1^)	23.6±6.2	15.7±12.2	1.6±1.1	0.3±0.2	0.1±0.1	12.9±1.9	4.4±0.4	7.3±13.8	0.8±0.3	0.3±0.4	14.8±1.3	2.4±0.8	0.6±0.8	0.1±0.0	0.1±0.0
Water temperature (°C)	36.1±4.6	28.2±5.9	29.3±7.2	28.4±6.9	28.8±7.0	33.8±1.7	27.8±3.3	26.8±2.6	26.3±3.3	26.6±3.2	32.1±2.3	28.0±1.9	27.2±1.4	27.0±1.1	26.1±1.1
NH_3_^−^ N (mg l**^−^** ^1^)	3.2±0.6	1.1±0.4	0.3±0.1	0.2±0.0	0.2±0.1	3.1±0.5	1.1±0.2	0.4±0.5	0.3±0.2	0.3±0.1	2.3±0.3	1.0±0.5	0.2±0.0	0.2±0.0	0.2±0.0
P-PO_4_^3−^ (mg l–1)	0.8±0.3	0.4±0.2	0.2±0.1	0.5±0.4	0.3±0.1	0.6±0.2	0.3±0.2	0.3±0.1	0.4±0.1	0.3±0.1	0.6±0.1	0.2±0.1	0.3±0.2	0.3±0.1	0.1±0.1
Dissolved oxygen (mg l**^−^** ^1^)	0.8±0.4	5.6±2.1	8.0±3.4	7.6±0.9	8.4±2.2	0.8±0.3	5.6±2.7	6.1±0.9	6.3±1.8	7.5±1.1	1.4±2.0	5.6±1.5	7.4±0.6	7.7±0.6	7.3±0.8
pH	7.7±0.2	8.2±0.1	8.3±0.2	8.5±0.1	8.7±0.1	7.9±0.1	8.1±0.1	8.2±0.1	8.4±0.2	8.7±0.1	8.0±0.3	8.1±0.1	8.3±0.2	8.8±0.1	9.1±0.1
Conductivity (mS cm**^−^** ^1^)	10.6±0.3	11.1±0.3	11.8±0.4	12.2±0.5	13.4±1.3	10.9±0.3	11.3±0.2	12.0±0.3	12.8±0.7	14.4±1.1	10.9±0.2	11.4±0.2	12.1±0.4	14.1±0.6	17.5±1.0
ORP (mV)	−287.6±23	85.7±89	103.0±84	117.6±65	110.8±74	−252.4±41	35.0±86	92.1±69	71.2±66	76.4±66	−125.3±236	66.4±74	84.5±68	71.1±62	67.6±60
SO_4_^2−^ (mg l**^−^** ^1^)	309.0±38	328.6±30	370.2±29	416.4±34	448.6±73	291.7±30	315.4±18	375.0±16	408.9±18	448.7±42	297.2±26	357.8±38	395.2±36	474.4±33	565.0±17
Boron (mg l**^−^** ^1^)	4.9±2.0	4.9±2.0	5.4±1.6	6.3±2.1	5.9±3.6	5.4±0.8	5.6±0.9	6.3±1.5	7.0±1.4	7.6±3.0	5.0±0.6	5.6±1.3	6.0±0.8	7.4±1.3	9.5±0.8
Vegetation	++	−	++	+	–	–	++	+	+	−	++	+	+	+	+

++ highly vegetated (75–100%); +moderately vegetated (25–75%); − sparsely vegetated (0–25%). ORP: Oxidation reduction potential.

### Fingerprinting Analysis

**Figure 2:**
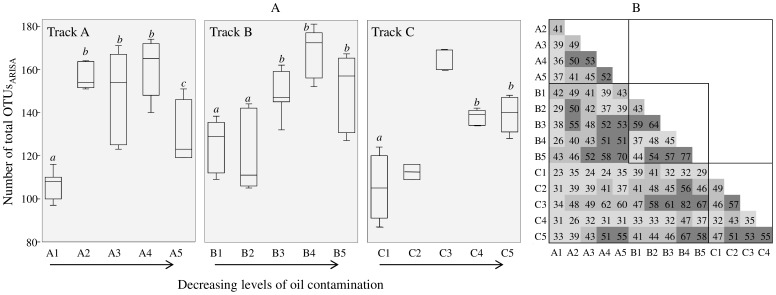
(A) ARISA-derived operational taxonomic (OTUs_ARISA_) numbers from all wetland mat samples. The median of each dataset is represented by middle line in each box. Common alphabetic superscripts denote no significant difference in OTU_ARISA_ number within the same track using Tukey’s test, the mats with less than 5 replicates were not statistically compared. (B) Percentage of OTUs_ARISA_ that are shared between the investigated mats.

The ARISA dataset yielded a pool of 353 distinct OTUs_ARISA_ (i.e. binned ARISA peaks) distributed among all samples, with average numbers ranging from 105 to 167 OTUs_ARISA_ (Fig. 2A). Average OTU_ARISA_ numbers in the mats from the sample points with highest oil contamination level (i.e. A1, A2, B1, B2, C1 and C2) were significantly (*P*<0.001) lower than in other sites (except in the case of A2). Pairwise comparison of presence/absence of OTUs_ARISA_ in the mats from different sample points was calculated to find out how many OTUs_ARISA_ are shared among the different communities (Fig. 2B). In all tracks, the number of shared OTUs_ARISA_ from each location did not exceed 60% in most cases (Fig. 2B). The highest number of shared OTUs_ARISA_ (i.e. 82%) was observed between B4 and C3 and the lowest (i.e. 23%) was between A1 and C1.

**Figure 3:**
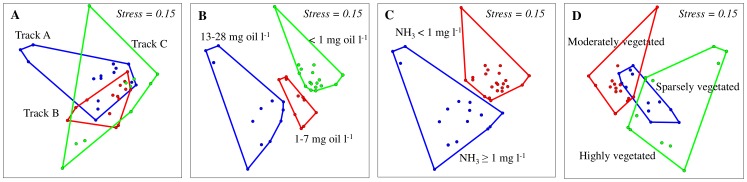
Non-metric multidimensional scaling (NMDS) ordination (based on a Bray-Curtis distance matrix) of ARISA fingerprints from the wetland mat samples based on analyzed subgroups. ANOSIM was used to test whether the bacterial communities are significantly different. A) depicts the ordination plot of investigated tracks, where ANOSIM showed no significance differences among the mats from different tracks (*R* = 0.15, Bonferroni corrected *P* = 0.07). B) ordination based on oil levels shows significant dissimilarities among the different subgroups (ANOSIM *R* = 0.52, Bonferroni corrected *P* = 0.0001). C) ordination based on ammonia concentrations shows the formation of two separate clusters (ANOSIM *R* = 0.54, Bonferroni corrected *P* = 0.0001). D) ordination plot based on plant densities shows a significant dissimilarity between the moderately vegetated and the highly vegetated mats (ANOSIM *R* = 0.43, Bonferroni corrected *P* = 0.0001) but not between the sparsely vegetated and the others mats.

When variations in bacterial community composition were visualized in a two-dimensional space using multivariate analyses of ARISA profiles (Fig. 3), bacterial communities of the three tracks clustered together (Fig. 3A) and were not significantly different (ANOSIM *R* = 0.15, Bonferroni-corrected *P* = 0.07). Out of the 353 identified OTUs_ARISA_, 165 OTUs_ARISA_ (i.e. 47%) were found common in the mats from the three tracks but 39, 31 and 12 OTUs_ARISA_ were ubiquitous residents of mats from Tracks A, B and C, respectively (S1A Figure). NMDS ordination based on the oil-contamination level placed the microbial communities in three separate clusters and this dissimilarity was supported by an ANOSIM *R* value of 0.52 (Bonferroni-corrected *P* = 0.002, Fig. 3B). The highest number of unique OTUs_ARISA_ was detected in the mats exposed to the lowest level of oil contamination (S1B Figure). The mats’ microbial communities were also significantly different when grouped according to NH_3_^–^N concentrations (ANOSIM *R* = 0.54, Bonferroni-corrected *P* = 0.0001, Fig. 3C, S1C Figure). Grouping the dataset according to the density of wetland plants in each cell showed that the mats’ microbial communities from highly and moderately vegetated tracks were significantly different from each other (ANOSIM *R* = 0.43, Bonferroni-corrected *P* = 0.0003, Fig. 3D) than from the mats with sparse vegetation, where they showed some overlap. The shared OTUs_ARISA_ between the mats from sparsely vegetated tracks and the mats from moderately and highly vegetated tracks were 51 and 27 OTUs_ARISA_, respectively (S1D Figure).

To obtain additional information on the environmental parameters that contributed to the variations of the microbial communities in the wetland mats, the relationships between ARISA fingerprints and all measured physical and chemical parameters (Table 1) were statistically evaluated (Table 2). All parameters, except Boron, significantly contributed to the variations in ARISA patterns. Among the most important determinants of microbial community composition were oil in water (11% of variance explained), NH_3_^–^N (11%), water temperature (9.6%), SO_4_^2−^ (9.8%) and pH (8.3%). Some covariation among factors was detected. Pure effects, which are effects because of a single factor taking all other factors into account, were generally not significant except for SO_4_^2−^ (Table 2).

**Table 2:** Effects of contextual parameters on variation in the wetland mat bacterial community structure.

Explanatory factors	Effect	*R^2^* (%)	*F*-ratio	*P* value
Oil in water	Total	11	2.75	0.001***
	Pure	0.6	1.08	*ns*
Water temperature	Total	9.6	2.45	0.001***
	Pure	3.3	1.45	0.061•
NH_3_^−^ N	Total	11	2.74	0.001***
	Pure	2.2	1.3	*ns*
P-PO_4_^3−^	Total	4.9	1.71	0.016*
Dissolved oxygen	Total	10.4	2.62	0.002**
pH	Total	8.3	2.27	0.001***
Conductivity	Total	6.1	1.91	0.009**
ORP	Total	8.6	2.32	0.003**
SO_4_^2−^	Total	9.8	2.51	0.001***
	Pure	4.8	1.66	0.025*
Boron	Total	6.6	1.98	*ns*

Total and pure effects (i.e. when controlling for all other factors of the analysis) of explanatory factors were calculated by using canonical redundancy analysis (RDA) models. The proportion of explained community variation is expressed as *R^2^* values. Significances of the respective *F*-ratios were tested by performing 1000 Monte Carlo permutation tests and are indicated by • marginally significant (*P*≤0.1), * significant (*P*≤0.05), ** very significant (*P*≤0.01), *** highly significant (*P*≤0.001), and *ns* when not significant (*P*>0.05).

### Pyrosequencing and Sequence Analysis

**Figure 4:**
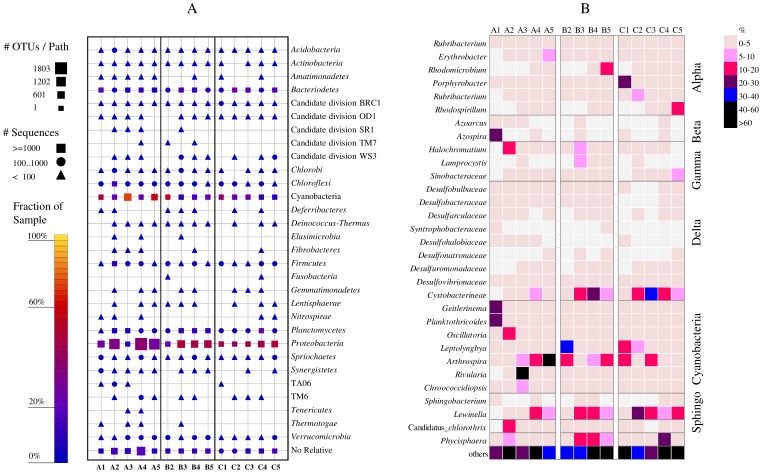
(A) Sequence frequency in the investigated wetland mat samples (B1 is missing) showing the major encountered bacterial classes. The shape of the symbol represents the number of sequences in each taxonomic bath, the size of the symbol represents the number of OTUs_0.03_ at deeper taxonomic levels with that taxonomic path and the color of the symbol indicates the relative frequency of the taxonomic path within the sample. (B) a heatmap representing a comparison of the relative abundance (% of total sequences) of the major bacterial genera and families in each bacterial class between the different samples. Only the distribution of sulfate reducing bacteria was indicated at the family level either due to the very low abundance of single genera or because of their unresolved taxonomy.

A total of 282,706 sequences of 16S rRNA gene were generated and ≤17% of the total number of sequences from each sample were unique without any relative (Table 3, Fig. 4). The number of detected OTUs_0.03_ ranged between 1386 and 3089. The highest bacterial richness, as determined by the number of OTUs_0.03_ and Chao1 index was highest in A2 and A4 samples and lowest in A1 and A3 samples (Table 2).

**Table 3:** Pyrosequencing and bacterial diversity estimators for the constructed wetland mat samples.

Sample ID	Total number of sequences	% of sequences with no relative	% of OTUs _0.03_ † Singletons^$^	Number of OTUs _0.03_ †	Chao-1
A1	40718	4.5	3.0	1386*	2882*
A2	20962	13.4	8.6	2939*	4811*
A3	43230	8.7	3.0	1397*	2585*
A4	23006	17.0	9.3	3089*	5510*
A5	35869	7.6	4.3	1848*	3265*
B2	14584	8.4	6.2	1867*	2943*
B3	12508	11.0	8.8	2348	3767
B4	12644	10.6	8.8	2445	3848
B5	11530	13.9	8.7	2232	3526
C1	15397	5.8	4.3	1418*	2210*
C2	10578	9.3	6.1	1455	2238
C3	16406	7.3	4.4	1503*	2399*
C4	13090	7.8	8.1	2198	3820
C5	12184	16.7	7.1	2048	3049

The diversity indices marked with an asterisk represent the average values calculated after performing three randomized sampling of the sequences to enable comparison among different samples.

† Operational taxonomic unit at 3% sequence dissimilarity; $ Singletons are sequences that were observed once.

Comparison between the sequence analysis data using QIIME and SILVA NG showed slight differences in the taxonomy of sequences, however the affiliation of major classes and genera was generally comparable. Here, we present only the results obtained from the SILVA NG pipeline, but restricted to the genera that were detected by both pipelines. Cyanobacteria constituted a major fraction of microbial communities of all mats with an abundance of 20–72%, 10–57% and 7–37% of total sequences of Track A, B and C, respectively. In A1 mats, most of the detected cyanobacterial sequences (44%) belonged to the genera *Geitlerinema* and *Planktothricoides* (Fig. 4B). These genera were replaced by *Oscillatoria* in A2 mats (15%), *Rivularia* (43%) and *Chroococcidiopsis* (9%) in A3 mats and by *Arthrospira* in A4 and A5 mats. In Track B and C mats, the majority of cyanobacterial sequences belonged to the genera *Leptolyngbya* and *Arthrospira*.

Among the proteobacterial classes, *Alpha*-, *Beta*- *Gamma*-, and *Deltaproteobacteria* were most frequently encountered with few sequences from *Epsilonproteobacteria* (≤1%). These classes exhibited variable distribution among the different mats (Fig. 4A). *Alphaproteobacteria* constituted between 3–40% of the total sequences, with higher numbers (>18%) in the mats from downstream locations (i.e. A4&5, B4&5 and C4&5). The obtained sequences were phylogenetically affiliated to purple non-sulfur bacterial sequences from the families *Rhodobacteraceae* and *Erythrobacteraceae*, and the genera *Rhodomicrobium*, *Porphyrobacter*, *Rubribacterium* and *Rhodospirillum* (Fig. 4B). Among the rare sequences within the *Alphaproteobacteria*, sequences related to nitrogen-fixing bacteria of the genera *Azospirillum*, *Rhizobium*, *Mesorhizobium* and *Bradyrhizobium* (each ≤1% of total sequences) were encountered.

*Betaproteobacteria* constituted ≤6% of the total sequences in all mats, except in the A1 mats, where it made up to 30% of the total sequences. 88% of these sequences belonged to the genus *Azospira* (Fig. 4B). Few sequences related to the nitrifying bacteria of the genera *Nitrospira* and *Nitrosomonas* were encountered. Sequences of the class *Gammaproteobacteria* exhibited the highest abundance in mats A2 and B3 (20 and 22% of the total sequences, respectively), while comprising between 2 to 10% of the total sequences in other mats. This class contained sequences that belonged to the purple sulfur bacterial genera *Halochromatium*, *Lamprocystis* and *Sinobacteraceae* as well as few other sequences of the nitrifying bacteria of the genus *Nitrosococcus.* The *Deltaproteobacteria* was least dominant in Track A mats (≤10%) but its abundance increased in Track B mats (max. 23%) and then again in Track C mats (max. 34%). The majority of sequences of this class belonged to the family *Cystobacterineae* and a few other sequences belonged to well-known sulfate-reducing bacterial genera, mainly *Desulfovibrio* and *Desulfobacula* (Fig. 4B).

Sequences belonging to the phylum *Bacteroidetes* were detected in all mats (Fig. 4A). The most detectable sequences of this group belonged to the genera *Lewinella* and *Sphingobacterium*. *Lewinella* sequences were detected in all mats, except A1 mats (Fig. 4B). In contrast, S*phingobacterium* was mainly detected in A1 mats (4% of the total sequences), and was almost absent in other mats (≤0.1%). *Chloroflexi* and *Planctomycetes* sequences composed ≤5% of total sequences in all mats, except in mat A2 where they constituted up to 19% and 7% of total sequences, respectively (Fig. 4A). About 85% of the sequences of the *Chloroflexi* group belonged to *Candidatus chlorothrix*, a filamentous anoxygenic photoautotroph isolated from Guerrero Negro hypersaline mats in Mexico [37] whereas most of the *Planctomycetes* sequences belonged to the genus *Phycisphaera*. The remaining less dominant groups (≤3% of total sequences) were distributed among several other groups (Fig. 4A) including *Verrucomicrobia*, *Acidobacteria*, *Actinobacteria*, *Chlorobi*, *Deinococcus*-*Thermus*, *Firmicutes* and *Spirochaetes*.

SIMPER analysis performed on pyrosequencing data revealed that the major species that contributed to the dissimilarity between bacterial communities were *Arthrospira platensis*, *Cystobacterineae* sp., *Leptolyngbya* sp., *Lewinella* sp., *Rivularia* sp. and *Phycisphaera* sp. (S1 Table). The contribution for any single species did not exceed 15.2%.

### Alkane Biodegradation by Track A Mats

Visual observations of the incubations showed a clear emulsification of oil with time and enhancement of cyanobacterial growth at the end of the experiment. GC analysis showed that all mats from Track A degraded 53–100% of C_12_ to C_30_ alkanes already after 6 weeks of incubation (S2 Table). Some of these alkanes, especially in the lighter fractions, completely disappeared from the GC histograms after 6 weeks (S2 Figure).

## Discussion

The constructed wetland cyanobacterial mats harbored a high diversity of microorganisms, as indicated by the OTUs_0.03_ richness and Chao index estimates, and microbial biogeography in these mats were influenced by both edaphic and biotic parameters. Microorganisms in these mats were capable of aerobically degrading petroleum compounds, thus contributing to the remediation of oil-polluted waters in the wetland. The microbial communities from Track A, B and C mats formed overlapping clusters in NMDS ordination (Fig. 3A). Nevertheless, the mats from different locations between the wetland inlet and outlet had clear variations in their microbial community composition. This is clearly reflected by the number of shared OTUs_ARISA_ among the different mats, which was <60% in most cases (Fig. 2B). The detected differences in bacterial composition were most likely because of the spatial variation in environmental parameters throughout the wetland. The mats had a large proportion of novel types since in each mat between 4.5% and 17% of the total sequences had no close match in publicly available 16S rRNA gene databases (Table 3). The uniqueness of these bacteria is probably a result of the harsh environmental conditions of the desert coupled with the long term and continuous exposure to oil contamination. Although we noted differences in taxa assignment when using different analysis pipelines, sequences assigned to the genera *Chroococcidiopsis*, *Deinococcus*-*Thermus*, *Halochromatium*, *Chloroflexi*, were detected by both employed pipelines in this study. These genera are known to include extremophilic species and their presence is consistent with the harsh environmental conditions of the wetland.

Oil concentration in water was one of the most determinant parameters for shaping up the microbial communities in the wetland mats as inferred from NMDS and RDA analyses. OTU_ARISA_ richness was significantly lower in the mats with higher levels of oil contamination, although all mats were still capable of degrading hydrocarbons. This could be due to the fact that while oil promotes the growth of hydrocarbon-degraders in the mats, it includes constituents that are toxic to other microorganisms ([38] and references therein). Thick viscous oil layers limit oxygen penetration and light penetration and could consequently affect the distribution of microorganisms, particularly the phototrophic types. This could explain the variable distribution of cyanobacteria in the mats from all tracks, especially in Track A, where oil concentrations strongly varied among the different locations (Table 1). Also, the dominance of the photosynthetic purple non-sulfur bacteria from the class *Alphaproteobacteria* in downstream sites (A4, 5, B4, 5 and C4, 5, Fig. 4B) could be attributed to the availability of more light, which is otherwise prevented by thick layers of oil as well as the dense vegetation in the upstream sites. Although petroleum compounds have been shown to drastically affect the growth and activity of photosynthetic organisms [39]–[43], cyanobacteria constituted a major fraction of the total sequences in most of the wetland mats. Their abundance, as also inferred from chlorophyll *a* concentrations (S3 Figure), was still high even in the mats with the highest levels of oil pollution. Field observations as well as experimental data have shown that oil-rich sediments supported the formation of dense cyanobacterial mats versus thin loose biofilms on oil-free sediments [44], [45]. This could be due to the following reasons: 1) the viscous oil provides a favorable substratum for the attachment and growth of cyanobacteria, 2) cyanobacteria produce excessive amounts of exopolymeric substances (EPS), in response to oil, which consolidate the sediment and 3) the growth of cyanobacteria might be triggered by the oil-degrading bacteria, which provide nutrients and locally released CO_2_, that can be immediately used for photosynthesis. In spite of the dominance of cyanobacteria in oil-polluted mats [15], [16], previous research demonstrated their inability to degrade hydrocarbons [18]. Instead, cyanobacteria played an indirect role in oil degradation by supporting the oil-degraders with oxygen produced via photosynthesis, nitrogen (N) fixed by nitrogen-fixing strains and simple organics produced by photosynthesis and fermentation [17], [18], [46], [47]. Indeed, the detection of high abundance of sequences related to the heterocystous nitrogen-fixing *Rivularia* genus of the order *Nostocales*, particularly in mat A3 (Fig. 4B), suggests a possible role of these cyanobacteria in enriching the wetland system with nutrients. Other detected cyanobacterial genera in the wetland mats were *Geitlerinema, Planktothricoides*, *Chroococcidiopsis*, *Leptolyngbya* and *Arthrospira* (Fig. 4B). While some of these genera have also been detected in other freshwater ecosystems [48]–[50], others, particularly the thin filamentous types, were observed elsewhere to overgrow dominant populations following catastrophic impacts and natural perturbation [51], [52].

The retrieved sequences from cyanobacteria, *Chloroflexi*, *Alpha-*, *Gamma-* and *Deltaproteobacteria* suggest the presence of carbon, nitrogen as well as sulfur cycle in the wetland mats. Microorganisms are known to play a vital role in nutrient cycling in aquatic ecosystems [53], hence some of the measured nutrients in the wetland could actually be a result of bacterial activities. Ammonia, which was found to significantly account for differences in the community composition of the wetland mats, is produced by N_2_ fixation but oxidized through nitrification. Both autotrophic (e.g. *Rivularia*) and heterotrophic genera (i.e. *Azospira Azospirillum*, *Rhizobium*, *Mesorhizobium* and *Bradyrhizobium*) of nitrogen fixers as well as a few sequences related to nitrifiers, were detected in the wetland mat samples (Fig. 4B). Previous studies demonstrated that N cycle processes such as N_2_ fixation, nitrification and denitrification are active in wetland ecosystems [54], [55]. *Rivularia* and *Azospira* were rarely found in other wetlands, although other types of heterocystous *Nostocales* cyanobacteria and heterotrophic diazotrophs were detected [13], [56], [57]. While autotrophic N_2_ fixation prevailed in oligotrophic and mesophilic wetlands, heterotrophic N_2_ fixation increased often after eutrophication [13], [50]. Therefore, the differences in N and P concentration in our wetland waters (Table 1) could explain the variable distribution of nitrogen-fixing microorganisms in the mats [58]. The activity of nitrogen-fixing microorganisms in microbial mats of other studies was shown to not only alleviate N limitation but also to support the growth and activity of oil-degrading heterotrophs [13], [46].

Most of the acquired sequences of *Alpha*- and *Gammaproteobacteria* belonged to purple non-sulfur and purple sulfur bacteria, respectively. These microorganisms, with their ability to perform anoxygenic photosynthesis, contribute, along with cyanobacteria, to the primary production of the wetland. Purple non-sulfur bacteria possess a versatile metabolism with the ability to grow as photoheterotrophs, photoautotrophs or chemoheterotrophs. Their growth mode in the wetland depends on several parameters including the degree of anoxia, availability of carbon source (CO_2_ for autotrophic growth or organic compounds for heterotrophic growth), and availability of light. Although purple non-sulfur bacteria were previously detected in oil-polluted mats, few strains could chemotrophically grow on hydrocarbons [59], [60]. We speculate that their dominance in the wetland is an indirect consequence of the high availability of light as well as CO_2_ and organics, resulting from the degradation of oil components. The detection of purple sulfur bacteria in the wetland mats is indicative of the anoxic conditions in deeper layers of the mats, where typically a sulfur cycle occurs. Surprisingly, very few sequences (≤0.5%) related to known sulfate-reducing bacteria were encountered (Fig. 4B). This constitutes a paradox, especially since it is well known that oil pollution (and high inputs of organic matter) stimulates sulfur cycle [61] and the measured sulfate levels in our wetland were quite high (Table 1). Rate measurements in various wetlands revealed high levels of sulfate reduction, often comparable to those measured in marine surface sediments [62], [63], thus pointing to the importance of this anaerobic degradation process in wetlands. Interestingly, the detection of few known sulfate reducing bacteria in our wetland is consistent with previous studies in other freshwater wetlands, where taxa of known sulfate-reducing bacteria were rarely detected or constituted a minor fraction (<1%) of the microbial community in 16S rRNA clone libraries [64]–[66]. Therefore, we speculate that, in our wetland mats, sulfate reduction is performed either by the detected rare members of sulfate-reducing bacteria or by novel yet-undescribed populations. This presence of rare or undescribed bacteria is conceivable, given the unique nature of this wetland ecosystem, having been artificially created within a harsh desert environment and supplied with moderately saline, oil-contaminated production water. Indeed, a stable isotope probing study of sulfate-reducing bacteria in a German wetland demonstrated that rare biosphere members played an active *in situ* role in the sulfur cycle [67]. Using *dsrAB* as marker genes, it was also demonstrated that the sulfate reducing community of 18 wetlands belonged to novel phylogenetic lineages that were unrelated to known sulfate reducing bacteria [62], [68]. These findings highlight the need to further investigate the diversity and activity of sulfate reducing bacteria in wetlands.

Vegetation in the wetland seems to be an important ecological driver of the distribution of microbial communities in the mats, as inferred from the NMDS ordination (Fig. 3D). Although, it is known that different plant species have distinct bacterial communities in their rhizosphere, it is not clear how vegetation could directly affect bacterial communities of the wetland mats. It is plausible that the effect of vegetation is indirect through the changes it induces in soil characteristics such as soil pH, soil texture and nutrients (C and N). Moreover, heavy vegetation causes shading, which reduces light penetration and decreases temperatures. The changes in all these parameters have a direct influence on mat communities. Indeed, our RDA analysis showed pH and temperature as important drivers controlling the mats’ bacterial community structure in our wetland (Table 2). Previous studies have demonstrated a clear impact of plant density and associated changes in soil characteristics on the composition of soil microbial communities [69]–[71].

An interesting feature of the wetland mats was the detection of a large proportion of myxobacteria from the genus *Cystobacterineae*, particularly in Track B and C mats (Fig. 4B). These microorganisms are regarded as typical soil microorganisms, but were reported in some microbial mats [72], [73] and in oil-polluted sediments [74]. Myxobacteria were considered as indicators of trophy level and contamination of water [75]. These microbes excrete hydrolytic enzymes and decompose complex biopolymers, feed on other prokaryotes and even eukaryotes and produce fruiting bodies and spores under unfavourable conditions [76]. These features make myxobacteria suitable to survive in our wetlands in which they can cope with the high organic loads of the water and the harsh conditions of the desert as well as feed on other organisms and plant detritus.

We conclude that the microbial distribution of the cyanobacterial mats in the studied constructed wetland system is driven by both biotic and environmental parameters and these mats play a vital role in the bioremediation of oil polluted waters in the wetland.

## Supporting Information

S1 Figure **Partitioning of OTUs_ARISA_ based on different tracks (A), oil levels (B), ammonia concentrations (C) and plant densities (D).**
(TIFF)

S2 Figure **GC/MS chromatograms showing the concentrations of crude oil fractions after 6 weeks of incubation of the wetland mats from Track A in the presence of additional crude oil versus a biotic control (oil+dead autoclaved mat).**
(TIFF)

S3 Figure **Chlorophyll** ***a*** **concentrations (in mg g^−1^) in the 15 investigated wetland mats.** The shown concentrations represent the average values obtained from triplicate samples and error.
(TIFF)

S1 Table **The contribution of particular bacteria to total dissimilarity (as percentages) between the bacterial communities of the mats in different tracks, at different oil contamination and NH_3_^–^N levels and at different plant densities using SIMPER (similarity of percentage) analysis.** The bacteria that contribute ≥5% to the dissimilarity are highlighted in grey.
(DOC)

S2 Table **Percentage concentration reduction (biodegradation) of alkanes C_12_–C_30_ (± standard deviation, n = 3) by the constructed wetland cyanobacterial mats from Track A after 6 weeks of incubation as detected by GC analysis.**
(DOC)
