# Supplementary figures and images for: Diversity, Distribution and Hydrocarbon Biodegradation Capabilities of Microbial Communities in Oil-Contaminated Cyanobacterial Mats from a Constructed Wetland
Source: PLoS One. 2014 Dec 16;9(12):e114570. doi: 10.1371/journal.pone.0114570 (PMC4267807; doi:10.1371/journal.pone.0114570)

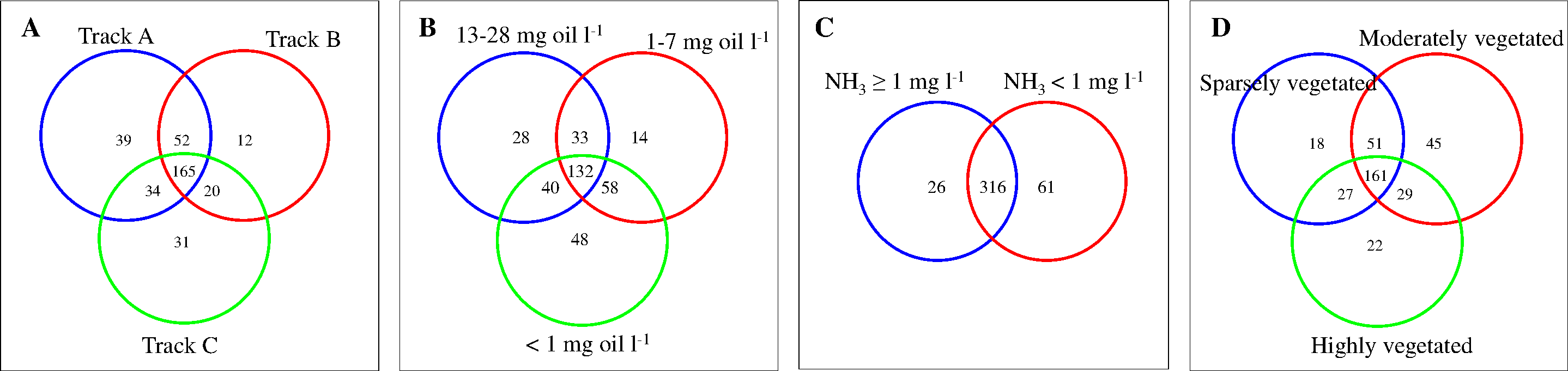

Supplement: S1 Figure — Partitioning of OTUsARISA based on different tracks (A), oil levels (B), ammonia concentrations (C) and plant densities (D). (TIFF) [file pone.0114570.s001.tiff]

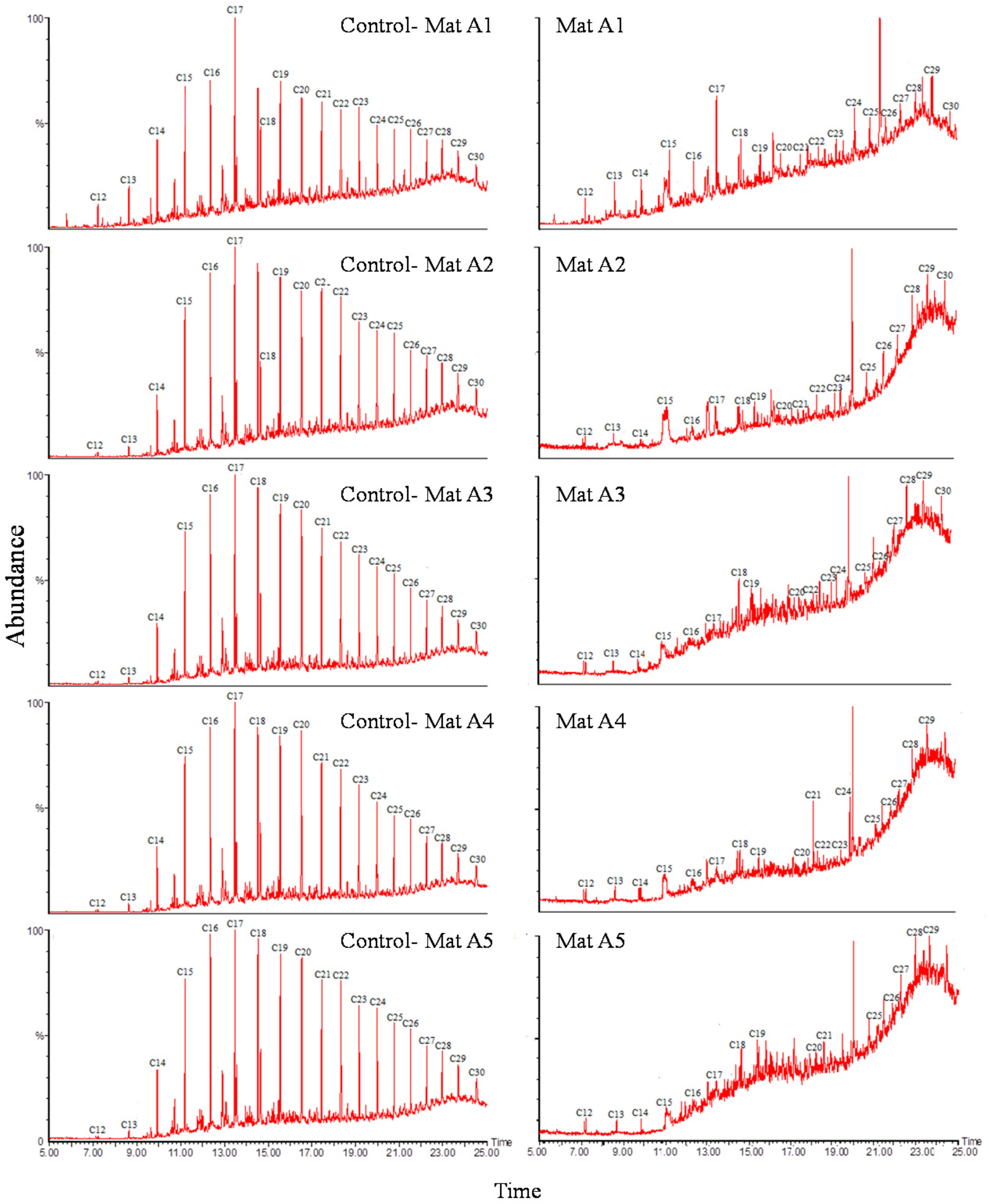

Supplement: S2 Figure — GC/MS chromatograms showing the concentrations of crude oil fractions after 6 weeks of incubation of the wetland mats from Track A in the presence of additional crude oil versus a biotic control (oil+dead autoclaved mat). (TIFF) [file pone.0114570.s002.tiff]

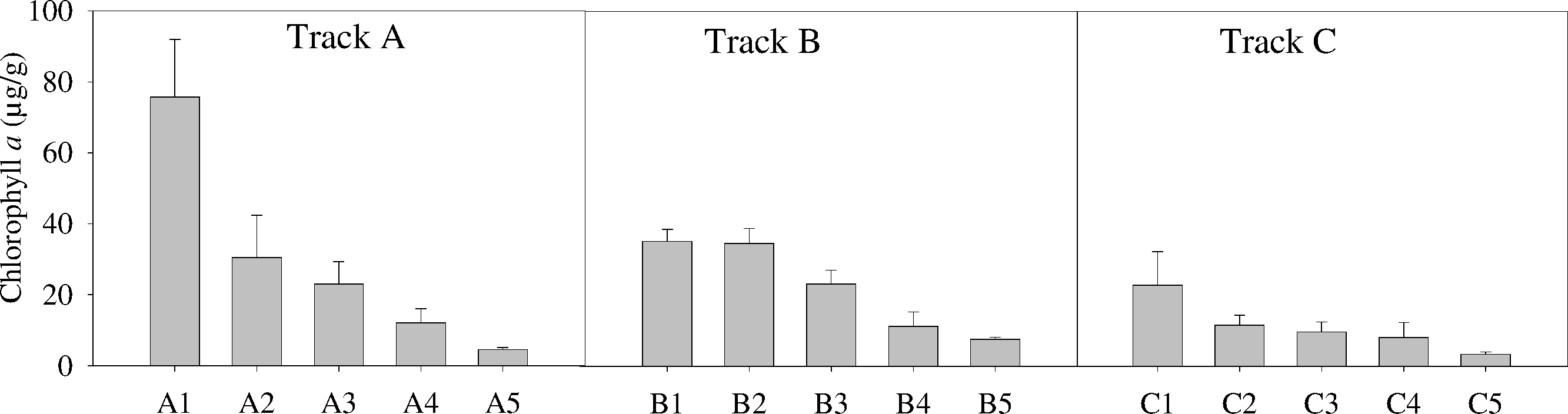

Supplement: S3 Figure — Chlorophyll a concentrations (in mg g−1) in the 15 investigated wetland mats. The shown concentrations represent the average values obtained from triplicate samples and error. (TIFF) [file pone.0114570.s003.tiff]
