# Supplementary material for: Diversity, Distribution and Hydrocarbon Biodegradation Capabilities of Microbial Communities in Oil-Contaminated Cyanobacterial Mats from a Constructed Wetland
Source: PLoS One. 2014 Dec 16;9(12):e114570. doi: 10.1371/journal.pone.0114570 (PMC4267807; doi:10.1371/journal.pone.0114570)
Supplement: S1 Table — The contribution of particular bacteria to total dissimilarity (as percentages) between the bacterial communities of the mats in different tracks, at different oil contamination and NH3–N levels and at different plant densities using SIMPER (similarity of percentage) analysis. The bacteria that contribute ≥5% to the dissimilarity are highlighted in grey. (DOC) [file pone.0114570.s004.doc]

| **Table S1** | | | | | | | | | | | | | |
| --- | --- | --- | --- | --- | --- | --- | --- | --- | --- | --- | --- | --- | --- |
| **Table S1.** The contribution of particular bacteria to total dissimilarity (as percentages) between the bacterial communities of the mats in different tracks, at different oil contamination and ammonia levels and at different plant densities using SIMPER (similarity of percentage) analysis. The bacteria that contribute ≥5% to the dissimilarity are highlighted in grey. | | | | | | | | | | | | | |
| Taxon | Species contribution to dissimilarity (%) | | | | | | | | | | | | |
|  | Sorted by track | | |  | By oil polution level | | |  | By NH_3_ level |  | By vegetation level | | |
|  | A&B | A &C | B&C |  | HP/MP | HP&LP | MP&LP |  | HN&LN |  | NV &MV | NV/HV | MV&HV |
| *Arthrospira platensis* | 10.4 | 9.6 | 4.7 |  | 3.2 | 8.0 | 9.9 |  | 7.2 |  | 15.2 | 12.7 | 3.7 |
| *Cystobacterineae* sp. | 7.2 | 9.7 | 10.6 |  | 6.0 | 8.3 | 8.9 |  | 7.9 |  | 12.1 | 1.3 | 11.0 |
| *Rivularia* sp. | 6.2 | 5.9 | 0.7 |  | 7.4 | 0.2 | 9.1 |  | 3.7 |  | 0.4 | 7.1 | 7.2 |
| *Leptolyngbya* sp. | 5.5 | 3.1 | 8.9 |  | 6.6 | 3.1 | 7.4 |  | 6.9 |  | 1.5 | 7.0 | 7.1 |
| *Phycisphaera* sp. | 4.6 | 4.0 | 6.6 |  | 2.1 | 4.0 | 5.0 |  | 4.1 |  | 4.6 | 2.6 | 4.4 |
| *Lewinella* sp. | 4.2 | 7.2 | 8.2 |  | 7.1 | 6.1 | 6.7 |  | 6.9 |  | 7.6 | 2.1 | 8.0 |
| *Azospira* sp. | 3.7 | 3.5 | 0.2 |  | 5.7 | 5.5 | 0.0 |  | 3.7 |  | 0.0 | 4.3 | 4.3 |
| *Planktothricoides* sp. | 3.5 | 3.1 | 1.4 |  | 4.7 | 4.7 | 1.3 |  | 3.6 |  | 0.4 | 4.1 | 4.3 |
| *Geitlerinema* sp. | 3.2 | 3.0 | 0.0 |  | 4.8 | 4.7 | 0.1 |  | 3.1 |  | 0.1 | 3.6 | 3.6 |
| *Candidatus Chlorothrix* sp. | 2.7 | 2.3 | 1.1 |  | 3.7 | 3.6 | 0.9 |  | 2.9 |  | 4.2 | 3.7 | 0.9 |
| *halochromatium* sp. | 2.7 | 1.7 | 2.1 |  | 3.1 | 2.6 | 1.9 |  | 2.2 |  | 3.4 | 2.7 | 0.9 |
| *Oscillatoria* sp. | 2.4 | 2.2 | 0.8 |  | 3.4 | 3.1 | 0.8 |  | 2.4 |  | 3.9 | 3.2 | 0.5 |
| *Porphyrobacter tepidarius* | 0.3 | 3.4 | 4.5 |  | 4.8 | 4.7 | 0.5 |  | 3.3 |  | 0.5 | 3.7 | 3.8 |
| Others | 43.4 | 41.3 | 50.2 |  | 37.3 | 41.6 | 47.5 |  | 42.3 |  | 46.0 | 41.9 | 40.3 |
