# Supplementary material for: Diversity, Distribution and Hydrocarbon Biodegradation Capabilities of Microbial Communities in Oil-Contaminated Cyanobacterial Mats from a Constructed Wetland
Source: PLoS One. 2014 Dec 16;9(12):e114570. doi: 10.1371/journal.pone.0114570 (PMC4267807; doi:10.1371/journal.pone.0114570)
Supplement: S2 Table — Percentage concentration reduction (biodegradation) of alkanes C12–C30 (± standard deviation, n = 3) by the constructed wetland cyanobacterial mats from Track A after 6 weeks of incubation as detected by GC analysis. (DOC) [file pone.0114570.s005.doc]

**Table S2**

| **Table S2**. Percentage concentration reduction (biodegradation) of alkanes C_12_-C_30_ (± standard deviation, n=3) by the constructed wetland cyanobacterial mats from Track A after 6 weeks of incubation as detected by GC analysis . | | | | | | | | | |
| --- | --- | --- | --- | --- | --- | --- | --- | --- | --- |
|
|
|
| Alkane |  | Mat A2 | | Mat A3 | | Mat A4 | | Mat A5 | |
| C_12_ | ND |  | ND |  | ND |  | ND |  | ND |
| C_13_ | 89±19 |  | 100±0 |  | ND |  | ND |  | ND |
| C_14_ | 98±3 |  | 100±0 |  | ND |  | ND |  | ND |
| C_15_ | 56±7 |  | 100±0 |  | 100±0 |  | 100±0 |  | 100±0 |
| C_16_ | 100±0 |  | 100±0 |  | 100±0 |  | 100±0 |  | 100±0 |
| C_17_ | 84±26 |  | 100±0 |  | 100±0 |  | 100±0 |  | 100±0 |
| C_18_ | 100±0 |  | 100±0 |  | 100±0 |  | 100±0 |  | 100±0 |
| C_19_ | 100±0 |  | 100±0 |  | 100±0 |  | 100±0 |  | 100±0 |
| C_20_ | 100±0 |  | 100±0 |  | 100±0 |  | 100±0 |  | 100±0 |
| C_21_ | 100±0 |  | 100±0 |  | 100±0 |  | 100±0 |  | 100±0 |
| C_22_ | 99±1 |  | 99±0 |  | 99±0 |  | 99±1 |  | 99±1 |
| C_23_ | 97±3 |  | 100±0 |  | 98±2 |  | 100±0 |  | 99±1 |
| C_24_ | 100±0 |  | 96±0 |  | 89±1 |  | 91±1 |  | 91±1 |
| C_25_ | 98±2 |  | 94±1 |  | 98±1 |  | 92±6 |  | 83±3 |
| C_26_ | 100±0 |  | 86±0 |  | 82±0 |  | 56±48 |  | 53±46 |
| C_27_ | 100±0 |  | 100±0 |  | 100±0 |  | 100±0 |  | 100±0 |
| C_28_ | 100±0 |  | 100±0 |  | 100±0 |  | 100±0 |  | 100±0 |
| C_29_ | 100±0 |  | 100±0 |  | 100±0 |  | 100±0 |  | 100±0 |
| C_30_ | 100±0 |  | 100±0 |  | 100±0 |  | 100±0 |  | 100±0 |
| ND: Not detected | | | | | | | | | |
